# Clinical Practice Guidelines of the Latin American Federation of Endocrinology for the use of vitamin D in the maintenance of bone health: recommendations for the Latin American context

**DOI:** 10.1007/s11657-024-01398-z

**Published:** 2024-06-08

**Authors:** Oscar Gómez, Claudia Campusano, Sonia Cerdas-P, Beatriz Mendoza, Amanda Páez-Talero, María Pilar de la Peña-Rodríguez, Alfredo Adolfo Reza-Albarrán, Pedro Nel Rueda-Plata

**Affiliations:** 1 Department of Psychiatry and Mental Health, Faculty of Medicine, Pontificia Universidad Javeriana, Bogotá, Colombia; 2 Facultad de Medicina de La Universidad de los Andes, Unidad de Endocrinología de La Clínica Universidad de los Andes, Sociedad Chilena de Endocrinología y Diabetes (SOCHED), Santiago, Chile; 3 Facultad de Medicina de la Universidad de Costa Rica, Servicio de Endocrinología del Hospital Cima San José, Asociación Costarricense de Endocrinología (ASCEND), San José, Costa Rica; 4 Clínica de Endocrinología y Metabolismo de La Facultad de Medicina de La República Oriental del Uruguay, Clínica de Endocrinología y Metabolismo del Hospital Manuel Quintela, Sociedad Uruguaya de Endocrinología y Metabolismo (SUEM), Montevideo, Uruguay; 5 Asociación Colombiana de Endocrinología, Diabetes y Metabolismo (ACE), Bogotá, Colombia; 6 Departamento de Endocrinología de La Facultad de Medicina de la Universidad Autónoma de Guadalajara (UAG), Presidenta Electa Para La Federación Latinoamericana de Endocrinología (FELAEN), Sociedad Mexicana de Nutrición y Endocrinología, Colegio Jalisciense de Endocrinología y Nutrición, Servicios Médicos De La Peña, SC Guadalajara, México; 7 Departamento de Endocrinología y Metabolismo del Instituto Nacional de Ciencias Médicas y Nutrición Salvador Zubirán, Universidad Nacional Autónoma de México, Sede Instituto Nacional de Ciencias Médicas y Nutrición Salvador Zubirán, Sociedad Mexicana de Nutrición y Endocrinología y Consejo Mexicano de Endocrinología, Ciudad de Mexico, México; 8 Federación Latinoamericana de Endocrinología (FELAEN), Universidad Nacional de Colombia, Asociación Colombiana de Endocrinología, Diabetes y Metabolismo (ACE), Bogotá, Colombia

**Keywords:** (MeSH): Bone diseases, Vitamin D deficiency, Dietary supplements, Health services, Practice guideline

## Abstract

**Introduction:**

These guidelines aim to provide evidence-based recommendations for the supplementation of Vitamin D in maintaining bone health. An unmet need persists in Latin American regarding the availability of clinical and real-world data for rationalizing the use of vitamin D supplementation. The objective of these guidelines is to establish clear and practical recommendations for healthcare practitioners from Latin American countries to address Vitamin D insufficiency in clinical practice.

**Methods:**

The guidelines were developed according to the GRADE-ADOLOPMENT methodology for the adaptation or adoption of CPGs or evidence-based recommendations. A search for high quality CPGs was complemented through a comprehensive review of recent literature, including randomized controlled trials, observational studies, and systematic reviews evaluating the effects of Vitamin D supplementation on bone health. The evidence to decision framework proposed by the GRADE Working Group was implemented by a panel of experts in endocrinology, bone health, and clinical research.

**Results:**

The guidelines recommend Vitamin D supplementation for individuals aged 18 and above, considering various populations, including healthy adults, individuals with osteopenia, osteoporosis patients, and institutionalized older adults. These recommendations offer dosing regimens depending on an individualized treatment plan, and monitoring intervals of serum 25-hydroxyvitamin D levels and adjustments based on individual results.

**Discussion:**

The guidelines highlight the role of Vitamin D in bone health and propose a standardized approach for healthcare practitioners to address Vitamin D insufficiency across Latin America. The panel underscored the necessity for generating local data and stressed the importance of considering regional geography, social dynamics, and cultural specificities when implementing these guidelines.

**Supplementary Information:**

The online version contains supplementary material available at 10.1007/s11657-024-01398-z.

## Introduction

Vitamin D deficiency has been implicated in decreased absorption of dietary calcium, leading to increased parathyroid hormone expression, subsequent bone resorption, and decreased skeletal mass [1]. Controversies still exist regarding vitamin D supplementation for maintaining bone health, particularly regarding optimal levels of 25-hydroxyvitamin D [25(OH)D] to start treatment [2]. Moreover, the absence of specific recommendations for Latin America highlights the need to account for the unique aspects of the region’s healthcare systems and the diversity of its communities [3, 4].

Dietary intake and safe sun exposure are traditionally the primary recommended sources of vitamin D for the general population [5]. However, in Latin America, challenges have been reported in attaining desired levels from these natural sources alone. A systematic review was carried out with the aim of shedding light on the extent of vitamin D deficiency in Latin America and the Caribbean. It was found that vitamin D deficiency in some countries could either be deemed non-existent as a public health problem (prevalence < 5%) or categorized as a mild public health issue (prevalence between 5 and 19.9%). However, the data revealed that in most countries, vitamin D deficiency was classified as a mild to moderate public health problem (prevalence between 20 and 39.9%), and in some cases, even as a severe public health issue (≥ 40%). Regarding dietary intake, a study carried out among Brazilian children and adolescents showing signs of stunting revealed that only 36% of these young individuals had an adequate intake of vitamin D. Similarly, a study in Costa Rica reported that the median intake of vitamin D for adolescents and young adults aged 12 to 20 years was 185 ± 7.6 IU, falling short of the 200 IU/day recommendation by the National Academy of Medicine (NAM, formerly the Institute of Medicine) [6].

Safe sun exposure is also recommended as a complementary natural method for achieving adequate vitamin D status, and variations in geographical conditions influencing vitamin D synthesis have been observed here as well. The D-SOL study (Interaction between Vitamin D Supplementation and Sunlight Exposure in Women Living in Opposite Latitudes) investigated the effects of vitamin D supplementation and sunlight exposure on vitamin D in Brazilian women living at high and low latitudes. A cross sectional analysis of the baseline data from this double-blind, randomized, placebo-controlled study demonstrated the significant influence of geographical location on Vitamin D status. More specifically, it was found that Brazilian women residing in England, situated at a higher latitude (51°N), exhibited a noticeably lower mean serum 25(OH)D concentration compared to those living in Brazil, a lower latitude (16°S). To achieve a 25(OH)D concentration of 30 ng/ml, the necessary UVB radiation exposure was determined to be 2.2 Standard Erythemal Dose (SED). The analysis of the complete data from the D-SOL study also revealed that a daily dose of 600 IU was effective in raising and maintaining adequate 25(OH)D levels regardless of latitude and sunlight exposure, as well as positively correlated to the baseline concentration of 25(OH)D [7, 8]. These findings underscore the significance of geographical location and associated sun exposure in dictating an individual’s vitamin D status and suggest that vitamin D supplementation could effectively improve vitamin D status, irrespective of geographical location and UVB exposure.

The Latin American Federation of Endocrinology (FELAEN), consistent with its mission, is dedicated to raising awareness about osteoporosis and taking a leading role in advancing the care of osteoporosis in the region [9] As part of its efforts, FELAEN released a position statement on the treatment of osteoporosis in postmenopausal women, wherein the use of vitamin D as a prevention and treatment strategy proved to be one the most difficult topics for reaching consensus [10]. To ensure an objective and evidence-based approach to this discussion, Clinical Practice Guidelines (CPG) were developed with the purpose of establishing recommendations for the use of vitamin D in maintaining bone health. The primary objective of the CPG is to provide evidence-based guidance specifically tailored to the needs and characteristics of the Latin American population, acknowledging the considerable variability within countries and even within regions of the same countries, including variations in sun exposure and dietary habits, which are the primary sources of vitamin D from natural sources. This aligns with FELAEN’s vision to take the initial steps toward harmonizing research, monitoring, and approaches to hypovitaminosis D in Latin America. During the development process, key topics were addressed such as the utility of measuring 25(OH)D levels, the optimal serum concentration for maintaining bone health, and the potential benefits and risks associated with vitamin D supplementation.

The recommendations presented in the CPG are aimed at the population aged 18 and above, including healthy adults, individuals with osteopenia, patients diagnosed with osteoporosis, and institutionalized older adults. These recommendations can be implemented by general practitioners and specialized physicians at all healthcare levels.

## Methods

To develop the CPG, a coordinating team was formed by authors PNR, APT, and MPDP, endocrinologists and members of FELAEN who served as thematic experts, along with OG, a medical epidemiologist who acted as the methodological adviser. The development was supported by an additional group of authors, who are members of national endocrinology associations from various Latin American countries that belong to FELAEN. The development of the CPG was based on the checklist described by the Guidelines International Network (GIN) and McMaster University [11], specifically employing the GRADE-ADOLOPMENT methodology, approach that optimizes resource management by leveraging the advantages of adopting, adapting, or developing novel recommendations for CPG. GRADE-ADOLOPMENT is built on GRADE Evidence to Decision framework and is aligned with a vision for better guidelines development, while allowing to provide locally contextualized guidance and recommendations for providers and stakeholders, and the opportunity to update the guidelines when new local evidence becomes available [12]. This approach provided an influential perspective on the current state of bone health and osteoporosis dynamics in the region [4]. The 12-step process encompasses various aspects of preparation and development of the final document, including its review and dissemination. Once the development protocol was agreed upon by the development group (DG), a provisional document was drafted, which defined the objectives and scope of the CPG, including the identification of healthcare professionals for whom they would be most useful and the target patient population. The document also formulated questions to be addressed in the CPG and defined methods for searching for and selecting sources of information to build the body of evidence used to develop recommendations.

## Body of evidence

The fundamental stage for formulating evidence-based recommendations presented in the CPG involved building a body of evidence and developing a decision-making process. This stage involved a thorough search using Latin American health regulatory authority websites as well as influential guideline developers, such as the Scottish Intercollegiate Guidelines Network (SIGN) and the United States Preventive Services Task Force (USPSTF), to identify relevant guidelines. Additional sources included the GIN CPG repository, Epistemonikos, the BIGG International Database of Grade Guidelines, and the Database of GRADE EtD’s and Guidelines. CPGs that aligned with the objectives of this project were selected based on the following inclusion and exclusion criteria:

- CPGs must be published from 2021 onwards to ensure the inclusion of the most current evidence.
- CPGs must provide evidence-based recommendations.
- CPGs must utilize a predefined methodology.
- CPGs must present the results of a quality assessment of the evidence.
- CPGs must provide an assessment of the evidence to generate recommendations.
- CPGs must not be adapted from other guidelines.

The CPGs that met these criteria were evaluated using the iCAHE tool for assessing the quality of CPGs [13].

The evidence from the selected CPGs was supplemented with searches for systematic reviews, randomized controlled trials (RCT), and non-randomized controlled trials (NRCT) published between January 2020 and June 2023. Primary studies were included if they had a minimum follow-up period of one year. Quality assessment was conducted using the AMSTAR 2 tool to evaluate systematic reviews, the ROB-2 tool to assess the risk of bias (RoB) in RCT, and the ROBINS-I tool to assess RoB in NRCT [14].

The body of the evidence was assessed using the GRADE methodology [15] and summarized using evidence tables (supplementary material) created in the free access version of the MAGIC authoring and publication platform (MAGICapp) [16]. The process for generating recommendations was based on a consensus reached through virtual sessions using the Nominal Group method [17].

## Key questions

Key questions were formulated to address routine measurement of 25(OH)D levels, the identification of individuals with low levels, treatment through supplementation to maintain bone health, and aspects related to these practices. These questions were discussed in virtual sessions among the members of the DG, prioritizing their alignment with guideline objectives, target populations, and clinical practice scenarios in Latin American countries.

1. Does screening for vitamin D deficiency contribute to maintaining bone health?
2. What are the risks of screening for vitamin D deficiency?
3. What is the optimal vitamin D status for maintaining bone health?
4. Does treating vitamin D deficiency contribute to maintaining bone health?
5. How should vitamin D supplementation be performed to achieve the optimal level for maintaining bone health?
6. What are the risks of treating vitamin D deficiency with supplements?

## Results

The process of searching and screening identified eight CPGs. (supplementary material), two of which were developed in Latin American countries (Mexico and Guatemala). Out of the eight screened, three CPGs published in 2021 were selected that aligned with the objectives of this project, met the inclusion and exclusion criteria, and had a high quality:

- ● National Osteoporosis Guidelines Group (NOGG): Clinical guideline for the prevention and treatment of osteoporosis [18].
- ● Scottish Intercollegiate Guidelines Network (SIGN): Management of osteoporosis and the prevention of fragility fractures [19].
- ● United States Preventive Services Task Force (USPSTF): Screening for vitamin D deficiency in adults [20].

A total of three systematic reviews [21–23] and three RCTs [24–26] were retrieved and evaluated for quality (supplementary material). The recommendations from the source CPGs were evaluated with respect to the Latin American context, considering any additional information included in the body of evidence. The DG discussed judgments and considerations made by the original panels.

## Clinical practice statements and recommendations

The recommendations developed for the CPG are presented in this section. Each recommendation is presented in a clear and actionable manner, indicating whether it was adopted, adapted, or developed de novo as per the GRADE-ADOLOPMENT approach [12]. The recommendations that were not adopted or adapted from the evaluated CPG are termed as developed, signifying the process of following the predefined methodology, which includes examining additional sources beyond the CPG. Additionally, clinical practice statements are included for situations where the available information was insufficient to form a recommendation. The accompanying text provides a detailed rationale for each statement and recommendation.

## Clinical practice statement

- The current evidence is insufficient to assess the balance of benefits and harms of universal screening for vitamin D deficiency in asymptomatic community-dwelling adults (adopted from USPSTF).

According to the review conducted by the USPSTF on the benefits and harms of screening for vitamin D deficiency, the current evidence remains insufficient to evaluate the risks associated with routine measurement of 25(OH)D levels in community-dwelling adults [27]. This conclusion aligns with their previous review, highlighting the ongoing uncertainty surrounding the potential benefits and harms of such screening practices [28]. The reviewers stated that they found no studies evaluating the benefits or potential harms of screening for vitamin D deficiency in community-dwelling adults, whether deficiency is defined as serum levels below 20 ng/ml or 30 ng/ml. They emphasized that additional research is needed to elucidate potential benefits. Furthermore, they propose that indirect evidence from well-conducted RCTs involving patients with vitamin D deficiency could help determine the optimal dosage and possible benefits, particularly in areas of high uncertainty, such as physical functioning, incidence of falls, and risks associated with intermittent high-dose regimens [29]. In Latin America, the practice of self-prescribing vitamins and other supplements without the guidance or supervision of a physician is prevalent [30]. Consequently, it is not uncommon to encounter individuals with either deficient or excessive serum levels of 25(OH)D. The DG advises that due consideration be given to this circumstance when assessing the suitability and potential risks associated with screening for vitamin D deficiency in Latin American patients.

## Clinical practice statement

- Providers should implement routine measurements of 25-hydroxyvitamin D levels in patients who are referred for the initial assessment of bone disease or abnormalities in mineral metabolism (developed).

The CPG for postmenopausal, glucocorticoid-induced, and male osteoporosis from the Spanish Society for Bone and Mineral Metabolism Investigation (SEIOMM) recommend a thorough blood and biochemical assessment for patients at risk of osteoporotic fractures. This biochemical analysis should be conducted regardless of the presence of a diagnosis of osteoporosis. It includes measuring the 25(OH)D levels in patients with conditions known to be associated with osteoporosis, such as hyperparathyroidism, hyperthyroidism, calcium deficiency, vitamin D deficiency, rheumatoid arthritis, and Cushing’s disease. Additionally, individuals undergoing treatment with glucocorticoids or aromatase inhibitors should also undergo this analysis. The same recommendation applies to individuals with tobacco use, regardless of concurrent alcohol consumption [31].

The panel considers that given the variability and access barriers to healthcare for Latin American patients at risk of compromised bone health, changes in mineral metabolism, and osteoporosis, routine referral visits at any care level could serve as an opportunity to evaluate the risk of osteoporotic fractures. Conclusions were drawn from the panelists' clinical experience and regional epidemiological data as reported in the 2021 Latin American Audit from the International Osteoporosis Foundation (IOF). In this audit, particular emphasis was placed on the growing population aged 50 years and older, recognizing its correlation with an elevated incidence of fragility fractures and the resultant burden and financial cost of osteoporosis [32]. The panel suggests that proactive strategies for early detection of risk factors for osteoporosis, such as vitamin D deficiency, could contribute to mitigating the disease burden on healthcare systems and improving quality of life for the Latin American population.

## Clinical practice statement

- Maintenance of a minimum 25-hydroxyvitamin D level of 20 ng/ml in the general population and in populations at risk of fractures is advised (developed).

The 2011 committee of the NAM commissioned two external systematic reviews and evaluated data judged as significant by the committee to determine the health outcomes influenced by vitamin D and calcium intake, as well as appropriate levels for safety. Bone health and measures of 25(OH)D and parathyroid hormone (PTH) were identified as relevant outcomes for evaluation. Upon reviewing the evidence, the committee found that 25(OH)D levels of 16 ng/ml meet the needs of roughly half the population. Levels of 20 ng/ml suffice for about 98% of the population. A 25(OH)D level of 20 ng/ml was found to be associated with maintaining femoral bone mass, and a level of 30 ng/ml was linked to low risk of hip fracture [33].

Although PTH levels were not considered adequate to establish the Dietary Reference Intake (DRI) of vitamin D by the NAM reviewers, it has been commonly used as a biomarker to estimate bone turnover due to availability, routine use, and modifiability via interventions such as vitamin D supplementation [34]. As such, a study that included a general population sample in Mexico, incorporating quotas for age groups 13 to 24, 25 to 50, and ≥ 51 years, reported the prevalence of low 25(OH)D levels and their correlation with PTH levels. Using the NAM cutoff point of 20 ng/ml for 25(OH)D, a biologically and statistically significant relationship with PTH levels in the Mexican population was identified, independent of major confounders such as age, weight, skin color, and diet. While most participants presented normal vitamin D status, those below 29 years of age exhibited a larger proportion of low concentrations. A Pearson correlation revealed a weak yet statistically significant negative relationship between 25(OH)D and PTH concentrations (*r* =  − 0.085, *p* = 0.039). A three-phase model emerged from a locally estimated scatterplot smoothing (LOESS), delineating two 25(OH)D thresholds at 19 ng/ml and 29 ng/ml:

- Phase 1 (25(OH)D < 19 ng/ml): inverse correlation with PTH (*p* < 0.01).
- Phase 2 (25(OH)D 20–29 ng/ml): gentle, non-significant direct correlation (*p* = 0.122).
- Phase 3 (25(OH)D > 29 ng/ml): no apparent relationship (*p* = 0.312).

Despite acknowledging limitations in the representativeness of the sample, the authors remark that the study provides valuable information on the interplay between vitamin D and PTH levels within a Latin American group [35].

The results of the analysis on PTH levels in the Mexican population are complementary to those of studies examining the relationship between vitamin D supplementation, 25(OH)D, and PTH levels. A systematic review evaluating the determinants of PTH level response to vitamin D supplementation indicated that although the intervention significantly reduces PTH levels with a pooled median difference (PMD) of − 8.0 pg/ml, this suppressive effect may require a prolonged duration (e.g., 12 months) and a high dose of supplementation [36]. These findings are consistent with recent randomized controlled trial (RCT) results demonstrating a greater decrease in PTH levels with higher doses of vitamin D3 (e.g., 48,000 IU monthly) [37]. Furthermore, a consistent inverse correlation was observed between measured PTH levels and those of total, bioavailable, and free 25(OH)D [38]. As a complementary observation, an RCT involving women of childbearing age assessed the response of intact PTH to vitamin D3 supplementation at doses of 600 IU, 1200 IU, and 4000 IU (accompanied by 500 mg calcium in all schemes). The researchers noted no suppression of this marker, suggesting that due to the high prevalence of vitamin D insufficiency at baseline, higher doses of vitamin D3 may be needed to achieve a suppressive effect over intact PTH [39].

The Mexican Health and Aging Study consisted of a survey on the burden of disease of an aging Mexican population obtained from a sample representative of the community-dwelling population aged 50 years and older in 2000. Using a subset of 1626 selected participants, it was found that after a mean follow-up of 162.1 weeks, the mortality rate was estimated at 12.6% for subjects that had a baseline 25(OH)D level below 15 ng/ml (22 deaths off a 174 subjects subsample). This was the highest rate of mortality among the subgroups analyzed, being reported at 2.9%, 3.1%, and 2.3% for subjects with baseline levels of 15 to 20 ng/ml, 20 to 29 ng/ml, and above 30 ng/ml, respectively (*p* < 0.001). Individuals with the lowest 25(OH)D were more commonly older, female, had lower scores on cognition, required more aid in basic activities of daily living (BADL), and had more chronic diseases. The difference in mortality rates (RR 5.421 95% CI [2.465–11.92]; *p* < 0.001) persisted after adjusting for age, sex, depression scores, dependency, number of chronic diseases, and cognitive impairment [40]. The findings of this study should be interpreted alongside the reanalysis of older data and recent analyses regarding the impact of vitamin D on mortality rates. Boullion and colleagues, in their review of the effects of vitamin D supplementation on health, compared the results from RCT and Mendelian randomization studies to emphasize the significance of context in commencing supplementation. They suggest that genetic predisposition to mortality might be more critical than vitamin D status in adequately supplemented populations, which contrasts with a potential modest benefit in reducing mortality risk among deficient individuals [41].

In Latin America, position statements from endocrinology associations have also considered the cutoff level of 30 ng/ml established by the Endocrine Society in its guidelines for the evaluation, treatment, and prevention of vitamin D deficiency [42], a document not included in the body of evidence for developing these CPG due to the defined date cut-off for assessing the most recent literature. However, the Endocrine Society CPG is currently undergoing updates and will undoubtedly be thoroughly discussed in the future.

The Brazilian Society of Endocrinology and Metabolism (SBEM) and the Brazilian Society of Clinical Pathology/Laboratory Medicine (SPBC) issued a consensus on reference values for 25(OH)D levels. They determined that levels between 20 and 60 ng/ml are sufficient for the general population under 65 years old and that levels between 30 and 60 ng/ml should ideally be maintained in older adults, individuals with a history of recurrent falls, those who have undergone bariatric surgery, pregnant women, patients on medication that interferes with vitamin D metabolism, and patients diagnosed with osteoporosis, secondary hyperparathyroidism, osteomalacia, type 1 diabetes mellitus, cancer, chronic kidney disease, or malabsorptive diseases. Deficiency levels were defined as below 20 ng/ml, and it was determined that levels exceeding 100 ng/ml should be evaluated for toxicity risk [43].

The USPSTF review identified a minimal difference in treatment effect depending on whether the RCTs enrolled community-dwelling participants with basal serum 25(OH)D levels less than 20 ng/ml versus those with levels less than 30 ng/ml [27].

For the cutoff of 20 ng/ml for a 25 (OH)D level, a systematic review of 96 studies reporting vitamin D status in various Latin American countries found an overall prevalence of vitamin D deficiency to be 34.76% (79 studies; 95% CI [29.68–40.21]; *I*^2^ = 99%). When a cut-off of 30 ng/ml was evaluated, the prevalence varied drastically, ranging from 7 to 98%. Significant differences in prevalence rates were related to age, sex, country, latitude, season, and year of study publication [22] (⨁◯◯◯).

The panel members, considering the positions adopted by the Endocrine Society and the societies that adhere to their proposed cutoff of 30 ng/ml of 25(OH)D, specifically as they relate to previous findings that suggest a possible benefit for groups such as postmenopausal women with osteoporosis, acknowledge that there is still a gap in information to establish the optimal cutoff to assess the need for vitamin D supplementation in this population. For this guideline, the DG accepted a value of 20 ng/ml as sufficient for maintaining bone health in healthy individuals and in individuals at risk of fracture in Latin America until sufficient local data is available to establish the most appropriate cutoff for the region.

The values of 25(OH)D identified in the literature as relevant are listed in Table 1.

**Table 1:** Cutoff values reported in the literature and their clinical relevance

25(OH)D cutoff (ng/ml)	Clinical relevance	Source
15	Higher mortality rates in a subset of a representative sample of community-dwelling adults from Mexico aged 50 years in 2000	[40]
16	Meets the needs of roughly half the population for bone health maintenance	NAM
20	Sufficient for about 98% of the population. Associated with maintaining femoral bone mass. Overall prevalence of vitamin D deficiency in Latin America is 34.76% (95% CI [29.68–40.21]). Inverse correlation with PTH in a Mexican sample	NAM
20 to 60	Adequate for the general population under 65 years old	SBEM/SPBC
30	Linked to a low risk of hip fracture. No significant reduction in risk above this level	Endocrine Society
30 to 60	Acceptable for adults, individuals with a history of recurrent falls, those who have undergone bariatric surgery, pregnant women, patients on medication that interferes with vitamin D metabolism	SBEM/SPBC
100	Should assess for toxicity risk at or above this value	SBEM/SPBC

*25(OH)D*, 25-hydroxy vitamin D3 (calcifediol); *NAM*, National Academy of Medicine; *95% CI*, 95% confidence interval; *PTH*, parathyroid hormone; *SBEM*, Brazilian Society of Endocrinology and Metabolism; *SPBC*, Brazilian Society of Clinical Pathology/Laboratory Medicine.

## Recommendation

- We suggest initiating vitamin D supplementation for the general population where deficiency is identified to maintain a 25(OH)D level above 20 ng/ml (developed, weak recommendation).

For community-dwelling adults with identified vitamin D deficiency, a slight net benefit in bone health has been reported in terms of lumbar spine and femoral neck Bone Mineral Density (BMD). However, little or no difference has been observed for hip BMD, spine fractures, femoral neck fractures, total hip fractures, or any fractures. The overall certainty of the evidence was deemed low concerning risk of bias in multiple domains as is explained below.

Data from the USPSTF systematic review, assessing bone health in community-dwelling adults treated for vitamin D deficiency, displayed a pooled Risk Ratio (RR) for any fracture of 0.84 (95% CI [0.58–1.21]), based on data from 2,186 participants in 6 studies with a follow-up period ranging from 12 weeks to 3.5 years. This equates to 9 fewer fractures per 1000 (95% CI [24 fewer–12 more]; ⨁⨁⨁◯). For hip fracture, the RR was 0.86 (95% CI [0.5–1.47]), based on data from 3,349 participants in 3 studies with a follow-up of 52 weeks to 3.5 years. This equated to 6 fewer fractures per 1000 (95% CI [22 fewer–21 more]; [27] ⨁⨁◯◯). Within the same review, a low incidence of kidney stones was observed across 10 RCTs, with the majority (80%) conducted among community-dwelling adults. Among all the studies, only one case of kidney stones was reported in a participant who received a daily dose of 800 IU of vitamin D without concurrent calcium supplementation [27].

The Cochrane systematic review evaluating the impact of vitamin D supplementation in healthy premenopausal women revealed that vitamin D alone does not significantly increase the BMD in the lumbar spine in this group. The studies included in these comparisons did not report identical outcomes and used different units of measure, so a pooled effect was not calculated. The risk of bias was low, with serious imprecision due to wide confidence intervals. The safety analysis demonstrated 61 fewer withdrawals for any reason per 1000 participants (CI 95% [126 fewer–44 more]) with vitamin D supplementation (⨁⨁⨁◯). The authors advocated for future studies to target populations predisposed to diseases related to bone metabolism or with diagnosed low bone mass or osteoporosis according to BMD measurements [23].

In a systematic review examining the effect of vitamin D3 supplementation in adult men and women aged 20 years and older without metastatic disease, notable increases in BMD at the lumbar spine (SMD 0.06; CI 95% [0.01–0.12]), and at the femoral neck (SMD 0.25; CI 95% [0.09–0.41]) were observed (⨁⨁◯◯). However, no difference was found in total hip BMD (SMD 0.13; 95% CI [-0.03–0.29]; ⨁⨁◯◯). The review identified a direct linear association between vitamin D3 supplementation and BMD at the femoral neck, lumbar spine, and total hip, as well as a nonlinear relationship with markers of bone turnover. Vitamin D3 supplementation was found to have a negative effect on forearm BMD. The results on bone density seemed to be affected by age, ethnicity, menopause status, baseline 25(OH)D concentration, and the frequency of vitamin D3 dosing. The most substantial improvement in BMD in response to supplementation was observed in individuals older than 65 years, those of non-white ethnicity, those with baseline 25(OH)D concentrations below 20 ng/ml, and those receiving daily supplementation rather than weekly or monthly. Substantial heterogeneity across studies regarding outcome ascertainment, type of population, and study design was found [21].

In the D-Health trial, investigators administered monthly doses of 60,000 IU of vitamin D to adults aged between 60 and 84 years, of whom 23.9% in the intervention group and 24.6% in the control group had a reported level of 25(OH)D below 20 ng/ml. After a follow-up period of 5 years with 20,326 participants, the authors reported minimal to no effect on non-vertebral fracture (HR 0.96; 95% CI [0.85–1.08]), major osteoporotic fracture (HR 1.00; 95% CI [0.85–1.18]), hip fracture (HR 1.11; 95% CI [0.86–1.45]), and any fracture (HR 0.94; 95% CI [0.84 – 1.06]; *p* = 0.32). A differential effect was noted for the predicted 25(OH)D concentration, with those at a level below 20 ng/ml at higher risk (HR 1.21; 95% CI [1.01–1.45]) for at least one osteoporotic fracture [26] (⨁⨁⨁⨁).

The VITAL study assessed the impact of vitamin D3 supplementation on fracture risk in generally healthy adults middle-aged and older, not specifically selected for vitamin D deficiency, low bone mass, or osteoporosis. The analysis involved data from 771 participants for a follow-up period of 2 years. The findings indicated that vitamin D3 supplementation did not significantly affect fracture risk. For this analysis, 18% of the subjects had a baseline 25(OH)D level below 20 ng/ml, for which no difference was seen in BMD between the vitamin D3, and the placebo groups as measured by DXA [24] (⨁⨁⨁◯). In an ancillary study of VITAL, it was determined that supplementation of vitamin D3 (at 2000 IU/d for 2 years) without concurrent calcium supplementation did not confer any significant benefits in terms of bone density or structure when compared to a placebo control. Specifically, vitamin D3 supplementation did not lead to an increase in BMD or prevent bone loss at the spine, hip, or whole body. Furthermore, vitamin D3 supplementation neither improved nor adversely impacted total, trabecular, or cortical BMD, cortical thickness, or bone strength at the radius or tibia. It is noteworthy that these effects were consistent across different baseline values of Body Mass Index (BMI), Fat Mass Index (FMI), age, and race/ethnicity, regardless of individual use of vitamin D supplements [25] (⨁⨁⨁⨁).

For this recommendation, the DG assessed the current evidence regarding the benefits of supplementing vitamin D for individuals identified as having insufficient 25(OH)D levels (< 20 ng/ml). Considering that a slight net benefit in bone health has been reported in terms of lumbar spine and femoral neck BMD and the emerging evidence of an increase in mortality rates for people with a baseline 25(OH)D level below 15 ng/ml in Latin American populations [40], the DG issued a weak recommendation in favor of vitamin D supplementation. Furthermore, the expert panel advises that in cases where a low serum value has been established, a supplementation regimen is warranted along with measurements of 25(OH)D levels to ensure the 25(OH)D level rise above the designated cutoff threshold of 20 ng/ml.

## Recommendation

- We recommend starting vitamin D supplementation for postmenopausal women as well as men aged 50 or above when vitamin D deficiency is identified or risk factors for vitamin D deficiency are present (adapted from NOGG, strong recommendation).

According to the authors of the NOGG CPG, it is crucial to ensure adequate dietary intake of key bone nutrients like calcium and vitamin D at all stages of life. They prefer dietary sources of calcium in clinical practice. However, if dietary calcium is insufficient and individuals are at risk of osteoporosis and/or fragility fractures, they suggest targeted supplementation of calcium combined with vitamin D. They note a potential increased risk of kidney stones with these supplements but not an increased incidence of cardiovascular disease or cancer (⨁⨁⨁⨁). Large, intermittent doses of vitamin D (e.g., ≥ 60,000 IU) are not advised due to reports of increased risk of fractures and falls [18] (⨁⨁⨁⨁).

## Recommendation

- We suggest considering the initiation of vitamin D treatment for frail, elderly individuals, such as nursing care residents who are at high risk of vitamin D deficiency, to reduce the risk of non-vertebral fractures (adopted from SIGN, weak recommendation).

In the formulation of their recommendation, the authors of the SIGN CPG assessed several meta-analyses, noting inconsistent results contingent on the specific RCTs incorporated into each analysis. They identified a singular RCT included in a meta-analysis, and that reported a minor but positive impact associated with calcium and vitamin D supplementation, specifically in reducing hip fracture risk in a particularly frail, older demographic. In terms of potential harm, calcium, and vitamin D combined supplementation was found to be associated with an increased risk of renal events, including renal insufficiency and calculi, compared to placebo or calcium supplements alone. Additionally, they highlighted a statistically significant association with an increased incidence of kidney stones compared to placebo. Regarding cardiovascular harms and mortality, the pooled RRs for coronary heart disease (CHD) events, all-cause mortality, myocardial infarction (MI), angina pectoris, and acute coronary syndrome (ACS) from relevant studies were not significantly different for those receiving calcium or combined calcium and vitamin D supplements compared to placebo (⨁⨁⨁⨁) [19].

## Recommendation

- We recommend starting vitamin D supplementation as an adjunct to anti-osteoporosis drug treatment if there is a suspicion or confirmed identification of vitamin D deficiency (adapted from NOGG, strong recommendation).

The authors of NOGG CPG point out that while many RCTs have tested the effects of either calcium alone, vitamin D alone, or the combination of both on reducing fracture risk, it is imperative that patients receiving anti-resorptive and anabolic osteoporosis drug therapies maintain adequate vitamin D status. This is a crucial point as it underlines the potential synergistic relationship between vitamin D and anti-osteoporosis medications in managing osteoporosis effectively (⨁⨁⨁⨁) [18].

## Recommendation

- We recommend selecting a suitable dosing regimen based on the availability of vitamin D supplements for individuals who require supplementation (developed, strong recommendation).

The Iberoamerican Society of Osteoporosis and Mineral Metabolism (SIBOMM) has formulated its position statement on vitamin D supplementation acknowledging the unique challenges posed by the diversity of the Latin American region and the barriers to obtaining vitamin D from natural sources. Not only is there a lack of access to foods rich in vitamin D, which is crucial for achieving optimal vitamin D status, the region also presents scenarios where solar exposure is insufficient for optimal synthesis of vitamin D despite its geographic advantage. SIBOMM emphasizes the importance of personalizing recommendations based on an individual’s health status, age, race, body weight, and geographical location, as well as dietary and cultural practices. For adults aged 19 to 70 years, SIBOMM suggests vitamin D supplementation ranging from 800 to 2000 IU/day. For those aged 70 years and above, they recommend a daily dosage of 1000 to 4000 IU, with the specific amount contingent on body weight and dietary intake. As part of a comprehensive approach, SIBOMM advises conducting a 25(OH)D level assessment three months after initiation of supplementation. An earlier test may be necessary in cases where vitamin D intoxication is suspected. This guidance underlines the importance of customization in the provision of vitamin D supplementation [44].

A systematic review and meta-analysis, which incorporated data from 24 studies and 1277 healthy participants, indicated a superior efficacy of cholecalciferol (vitamin D3) over ergocalciferol (vitamin D2) in addressing vitamin D deficiency. Specifically, cholecalciferol demonstrated a more pronounced effect in enhancing total 25(OH)D levels with a mean difference of approximately 6.28 ng/ml (95% CI [3.79–8.79]) and in reducing PTH levels. This advantage of cholecalciferol remained consistent across varying participant demographics, dosages, and supplementation methods. It was also found that the difference in efficacy between cholecalciferol and ergocalciferol was smaller at lower doses, with the average daily dose identified as a significant predictor of effect size [45]. For consideration, the results of a meta-analysis on the effect of vitamin D2 supplementation indicated that ergocalciferol supplementation significantly increases total vitamin D, 25(OH)D2, and 1,25(OH)D concentrations but decreases 25(OH)D3 levels. Subgroup analyses revealed that higher doses of vitamin D2 (> 2000 IU/day), shorter treatment durations (≤ 12 weeks), older age (≥ 60 years), and lower baseline 25(OH)D concentrations (< 20 ng/mL) were associated with more significant increases in total vitamin D levels. These findings emphasize the importance of tailoring vitamin D2 supplementation based on patient characteristics and treatment parameters [46]. In Latin America, ergocalciferol is only accessible in Argentina, Cuba, México, Panamá, and Perú [32]. The advice of the panel is to offer ergocalciferol where obtainable, particularly as an alternative for vegan patients.

In the VITAL-DKD study, which compared supplementation with 2000 IU of vitamin D to placebo in community-dwelling older adults, the baseline showed a positive, non-linear relationship between total serum vitamin D and total serum 25(OH)D concentrations. After adjusting for supplementation, an increase of approximately 11.7 ng/ml in serum vitamin D and roughly 13.4 ng/ml in serum 25(OH)D was noted. When participants with baseline 25(OH)D levels below 20 ng/ml were compared with those with levels at or above 20 ng/ml, the increase in serum vitamin D following supplementation was less pronounced in those with lower baseline levels (around 6.3 ng/ml compared to approximately 12.5 ng/ml; interaction *p* = 0.02). On the other hand, those with baseline levels below 20 ng/ml experienced a more substantial rise in serum 25(OH)D post-supplementation (around 19.2 ng/ml compared to approximately 12.3 ng/ml; interaction *p* = 0.05; ⨁⨁⨁⨁) [47]. The findings from this study affirm the efficacy of a commonly used dosing scheme for restoring adequate 25(OH)D levels.

Calcifediol represents another form of vitamin D available for supplementation, offering several distinct advantages over vitamin D3, especially in specific clinical situations. Calcifediol is one step closer to becoming biologically active, which provides it with pharmacokinetic properties that prove advantageous under circumstances like malabsorption, liver impairment, and obesity. Unlike vitamin D3, calcifediol’s intestinal absorption does not rely on bile acids and micelle formation, making it a superior choice for patients with conditions leading to fat malabsorption such as celiac disease, pancreatic insufficiency, or biliary cirrhosis. In these instances, while vitamin D3’s intestinal absorption is significantly compromised, calcifediol’s absorption remains relatively unaffected. Comparative clinical trials have shown that calcifediol at doses comparable to those of vitamin D3 induces a more rapid and larger increase in serum 25(OH)D concentrations. In terms of potency, calcifediol is between three and six times more potent than vitamin D3, a factor that may be beneficial when a rapid increase in vitamin D is needed [48].

A double‐blind, multicentric RCT enrolled 303 patients, with 298 included in the intention-to-treat (ITT) population allocated into three groups. One group received calcifediol 0.266 mg/month for 12 months, the second group received calcifediol 0.266 mg/month for four months followed by placebo for eight months, and the third group received cholecalciferol 25,000 IU/month for 12 months. The results showed that calcifediol was superior to cholecalciferol in achieving the 20 ng/ml threshold at both month 1 and month 4. At month 1, 59.0% of patients on calcifediol achieved the threshold compared to 34.0% on cholecalciferol. By month 4, these percentages increased to 81.0% and 72.4% for calcifediol and cholecalciferol, respectively, although the difference was not statistically significant. The most considerable disparity between the two drugs was observed in the mean change in serum 25(OH)D levels after the first month of treatment, with a mean change of 9.7 ± 6.7 ng/ml in patients treated with calcifediol versus 5.1 ± 3.5 ng/ml in patients treated with cholecalciferol. No significant treatment-related safety issues were reported in any study groups [49]. Calcifediol may be preferable for supplementing patients with malabsorption syndromes, including short bowel syndrome, and those undergoing bariatric surgery. It may also be preferable for patients taking medications that interfere with fat absorption [50].

In the process of selecting the suitable dosing scheme for supplementation, the expert panel considered the following: Latin America exhibits considerable diversity in the availability of pharmaceutical forms and concentrations, as well as access to vitamin D supplements. Furthermore, even with the adoption of an appropriate regimen based on availability and anticipated elevation in 25(OH)D levels in accordance with the chosen compound, individual factors can introduce variability in response to supplementation. For instance, with the growing prevalence of obesity in Latin America [51], there is a critical need for research investigating the impact of supplementation, accounting for factors such as the sequestering effect of adipose tissue on vitamin D forms [52]. There is currently insufficient information to determine whether the optimal strategy for this situation should involve dosage adjustments, weight reduction interventions, or a combination of both. The dosing schemes suggested by the panel are presented in Table 2. The indications suggested in the table are based on the consensus reached by the panel, drawing upon their collective experience in treating patients across various countries in Latin America.

**Table 2:** Recommended dosing schemes for supplementing vitamin D

Presentation	Loading dose^	Loading dose duration	Maintenance dose	Time to measure response
Cholecalciferol	4,000 to 5,000 IU/day	2 to 3 mos^‡^	800 to 1000 IU/day^†^	3 to 6 mos after initiating supplementation^+^
Cholecalciferol	25,000 to 50,000 IU/week	2 to 3 mos^‡^	5,000 to 10,000 IU/week^†^	3 to 6 mos after initiating supplementation
Cholecalciferol^^^	50,000 to 100,000/mo	3 mo	800 to 1000 IU/day^†^	3 to 6 mos after supplementation
Cholecalciferol^^^	50,000 to 100,000 as single dose	–	800 to 1000 IU/day^†^	3 to 6 mos after supplementation
Calcifediol	0.266 mg/every two weeks/3 mos	3 mos	0.266 mg/mo	3 to 6 mos after initiating supplementation

^: Use loading dose if 25(OH)D below 12 ng/ml (severe deficiency). †: according to risks for bone disease. Low doses preferred for low-risk patients. ‡: 2 mos if 25(OH)D is at 10 ng/ml to 20 ng/ml, and 3 mos if < 10 ng/ml. ^^^: Use these dosing schemes if rapid increase in 25(OH)D level is desirable. + : 3 months if started at loading dose. 6 months if low or maintenance dose is used. IU, international units; mo, month; mos, months.

## Discussion

In developing the CPG, the DG stressed the complex role of vitamin D in bone health. Determinations derived from the evidence reviewed for this work demonstrate a variable pattern in the impact of vitamin D supplementation on bone mineral density, fracture risk, and overall bone health. While some studies indicate a minor beneficial effect of vitamin D supplementation on bone mineral density, particularly in individuals with identified vitamin D deficiency or risk factors for it, others suggest no significant impact in healthier populations.

A crucial point to consider is the differences among health systems in the Latin American countries [53]. These inform the availability of preventive and therapeutic options for maintaining bone health and treating bone diseases across the region. Adding to this complexity is the diverse geographic landscape and corresponding variation in sunlight exposure present across the region [3]. Despite a significant portion of Latin America encompassing the equatorial region, where sunlight exposure and resulting vitamin D synthesis might be expected to be optimal, there are unique regional factors that might contribute to vitamin D deficiency. For instance, a notable number of cities are located in the Andean region [54], where high altitudes and cooler climates could lead to reduced sunlight exposure and vitamin D synthesis. Furthermore, countries outside the tropical zone experience seasonal variations in sunlight exposure [55, 56], which can significantly influence vitamin D status.

Moreover, the socioeconomic realities of Latin America need to be considered. Individuals living in urban areas, particularly in low-income communities, may have limited access to outdoor spaces. Indoor occupations, air pollution, cultural practices, and safety concerns may further restrict sunlight exposure [57, 58], thereby affecting the endogenous production of vitamin D. In addition, economic barriers may limit access to vitamin D-rich foods or supplements, particularly among underserved populations [59]. These socioeconomic factors can exacerbate the risk of vitamin D deficiency, even with the geographical advantage of certain regions.

The development of these Clinical Practice Guidelines was limited by the scarcity of experimental data from Latin America concerning the effects of vitamin D alone on bone health, and the unique demographic, nutritional, and sunlight exposure characteristics of the region may potentially modulate these. While the guidelines provide valuable insights, they also underscore the urgent need for region-specific research and health system strengthening in Latin America. They highlight the importance of tailoring bone health interventions to the unique challenges and needs of the region to optimize health outcomes.

## External review and quality assurance

The final version of this document was agreed upon by the DG after a preliminary version was submitted for open review through the FELAEN website. The manuscript was drafted following the RIGHT-Ad@pt Checklist, which is based on the methodology of the Reporting Items for Practice Guidelines in Healthcare (RIGHT) Working Group.

## Supplementary Information

Below is the link to the electronic supplementary material.

Supplementary file1 (DOCX 64 KB)

## Data Availability

The guidelines presented in this paper were developed based on existing literature, clinical practice guidelines, and consensus among the authors. No primary data were collected or utilized in the development of these guidelines. All data used to inform the development of the guidelines, including relevant studies, clinical trials, and meta-analyses, are openly available in the referenced sources cited in the report.
