# Supplementary material for: Clinical Practice Guidelines of the Latin American Federation of Endocrinology for the use of vitamin D in the maintenance of bone health: recommendations for the Latin American context
Source: Arch Osteoporos. 2024 Jun 8;19(1):46. doi: 10.1007/s11657-024-01398-z (PMC11162390; doi:10.1007/s11657-024-01398-z)
Supplement: Supplementary file 1 — Supplementary file1 (DOCX 64 KB) [file 11657_2024_1398_MOESM1_ESM.docx]

**SUPPLEMENTARY MATERIAL**

[CLINICAL PRACTICE GUIDELINES SCREENED 2](#_Toc151731258)

[QUALITY ASSESSMENT 3](#_Toc151731259)

[CLINICAL PRACTICE GUIDELINES 3](#_Toc151731260)

[Vitamin D Deficiency in Adults: Screening 3](#_Toc151731261)

[Management of osteoporosis and the prevention of fragility fractures 4](#_Toc151731262)

[Clinical guideline for the prevention and treatment of osteoporosis 4](#_Toc151731263)

[SYSTEMATIC REVIEWS – METANALYSIS 5](#_Toc151731264)

[Calcium and vitamin D for increasing bone mineral density in premenopausal women 5](#_Toc151731265)

[Prevalence of vitamin D deficiency in South America: a systematic review and meta-analysis 7](#_Toc151731266)

[Effect of supplemental vitamin D3 on bone mineral density - a systematic review and meta-analysis 9](#_Toc151731267)

[RANDOMIZED CONTROLED TRIALS 11](#_Toc151731268)

[The effect of monthly vitamin D supplementation on fractures: a tertiary outcome from the population-based, double-blind, randomised, placebo-controlled D-Health trial 11](#_Toc151731269)

[Supplemental Vitamin D and Incident Fractures in Midlife and Older Adults 13](#_Toc151731270)

[Effects of Supplemental Vitamin D on Bone Health Outcomes in Women and Men in the VITamin D and OmegA-3 TriaL (VITAL) 16](#_Toc151731271)

[EVIDENCE TABLES 19](#_Toc151731272)

# CLINICAL PRACTICE GUIDELINES SCREENED

| Guideline | Year | Country | Organism  Society | Evidence based | Rates evidence | Access to evidence assessment | Adapted or non-primary development | Selected |
| --- | --- | --- | --- | --- | --- | --- | --- | --- |
| Manejo clínico de la osteoporosis | 2019 | Guatemala | IGSS | Yes | No | No | Uncertain | No |
| Diagnóstico y tratamiento de osteoporosis en mujeres posmenopáusicas | 2018 | Mexico | IMSS | Yes | No | No | Yes | No |
| Management of Osteoporosis | 2022 | Malaysia | MOH | Yes | Yes | No | No | No |
| Clinical practice guideline of postmenopausal, glucocorticoid-induced and male osteoporosis | 2022 | Spain | SEIOMM | Yes | Yes | No | Uncertain | No |
| Vitamin D Deficiency in Adults: Screening | 2021 | USA | USPSTF | Yes | Yes | Yes | No | Yes |
| Management of osteoporosis and the prevention of fragility fractures | 2021 | Scotland | SIGN | Yes | Yes | Yes | No | Yes |
| Clinical guideline for the prevention and treatment of osteoporosis | 2021 | UK | NOGG | Yes | Yes | Yes | No | Yes |
| Management of Postmenopausal Osteoporosis | 2022 | USA | ACOG | Yes | Yes | Yes | Yes | No |

IGSS: Instituto Guatemalteco de Seguridad Social. IMSS: Instituto Mexicano del Seguro Social. MOH: Ministry of Health. SEIOMM: Spanish Society for Bone and Mineral Metabolism Investigation. USPSTF: United States Preventive Services Task Force. NOGG: National Osteoporosis Guideline Group. SIGN: Scottish Intercollegiate Guidelines Network.

# QUALITY ASSESSMENT

## CLINICAL PRACTICE GUIDELINES

### Vitamin D Deficiency in Adults: Screening

**Guideline producer:** United States Preventive Services Task Force.

**Link:** <https://www.uspreventiveservicestaskforce.org/uspstf/recommendation/vitamin-d-deficiency-screening>

| **Availability** | **Score** |
| --- | --- |
| Is the guideline readily available in full text? | 1 |
| Does the guideline provide a complete reference list? | 1 |
| Does the guideline provide a summary of its recommendations? | 1 |
| **Dates** |  |
| Is there a date of completion available? | 1 |
| Does the guideline provide an anticipated review date | 0 |
| Does the guideline provide dates for when literature was included? (1) | 1 |
| **Underlying Evidence** |  |
| Does the guideline provide an outline of the strategy they used to find underlying evidence? | 1 |
| Does the guideline use a hierarchy to rank the quality of the underlying evidence? | 1 |
| Does the guideline appraise the quality of the evidence which underpins its recommendations? | 1 |
| Does the guideline link the hierarchy and quality of underlying evidence to each recommendation? | 1 |
| **Guideline developers** |  |
| Are the developers of the guideline clearly stated? | 1 |
| Does the qualifications and expertise of the guideline developer(s) link with the purpose of the guideline and its end users? | 1 |
| **Guideline purpose and users** |  |
| Are the purpose and target users of the guideline stated? | 1 |
| **Ease of use** |  |
| Is the guideline readable and easy to navigate? | 1 |
| **Score** (total/14)*100 | 92,8 |

### Management of osteoporosis and the prevention of fragility fractures

**Guideline producer:** Scottish Intercollegiate Guideline Network

**Link:** <https://www.sign.ac.uk/media/1812/sign-142-osteoporosis-v3.pdf>

| **Availability** | **Score** |
| --- | --- |
| Is the guideline readily available in full text? | 1 |
| Does the guideline provide a complete reference list? | 1 |
| Does the guideline provide a summary of its recommendations? | 0 |
| **Dates** |  |
| Is there a date of completion available? | 1 |
| Does the guideline provide an anticipated review date | 1 |
| Does the guideline provide dates for when literature was included? (1) | 1 |
| **Underlying Evidence** |  |
| Does the guideline provide an outline of the strategy they used to find underlying evidence? | 1 |
| Does the guideline use a hierarchy to rank the quality of the underlying evidence? | 1 |
| Does the guideline appraise the quality of the evidence which underpins its recommendations? | 1 |
| Does the guideline link the hierarchy and quality of underlying evidence to each recommendation? | 1 |
| **Guideline developers** |  |
| Are the developers of the guideline clearly stated? | 1 |
| Does the qualifications and expertise of the guideline developer(s) link with the purpose of the guideline and its end users? | 1 |
| **Guideline purpose and users** |  |
| Are the purpose and target users of the guideline stated? | 1 |
| **Ease of use** |  |
| Is the guideline readable and easy to navigate? | 1 |
| **Score** (total/14)*100 | 92,8 |

### Clinical guideline for the prevention and treatment of osteoporosis

**Guideline producer:** National Osteoporosis Guideline Group UK.

**Link:** <https://www.nogg.org.uk/full-guideline>

| **Availability** | **Score** |
| --- | --- |
| Is the guideline readily available in full text? | 1 |
| Does the guideline provide a complete reference list? | 1 |
| Does the guideline provide a summary of its recommendations? | 1 |
| **Dates** |  |
| Is there a date of completion available? | 0 |
| Does the guideline provide an anticipated review date | 1 |
| Does the guideline provide dates for when literature was included? (1) | 1 |
| **Underlying Evidence** |  |
| Does the guideline provide an outline of the strategy they used to find underlying evidence? | 0 |
| Does the guideline use a hierarchy to rank the quality of the underlying evidence? | 1 |
| Does the guideline appraise the quality of the evidence which underpins its recommendations? | 1 |
| Does the guideline link the hierarchy and quality of underlying evidence to each recommendation? | 1 |
| **Guideline developers** |  |
| Are the developers of the guideline clearly stated? | 1 |
| Does the qualifications and expertise of the guideline developer(s) link with the purpose of the guideline and its end users? | 1 |
| **Guideline purpose and users** |  |
| Are the purpose and target users of the guideline stated? | 1 |
| **Ease of use** |  |
| Is the guideline readable and easy to navigate? | 1 |
| **Score** (total/14)*100 | 85,8 |

## SYSTEMATIC REVIEWS – METANALYSIS

### Calcium and vitamin D for increasing bone mineral density in premenopausal women

Méndez-Sánchez L, Clark P, Winzenberg TM, Tugwell P, Correa-Burrows P, Costello R. Calcium and vitamin D for increasing bone mineral density in premenopausal women. The Cochrane database of systematic reviews. 2023;1(1):CD012664.

| \| Calcium and vitamin D for increasing bone mineral density in premenopausal is a **Moderate quality review** \| \| \| --- \| --- \| \| **1. Did the research questions and inclusion criteria for the review include the components of PICO?** \| Yes \| \|  \| \| |
| --- | --- | --- | --- | --- | --- | --- |
| \| **2. Did the report of the review contain an explicit statement that the review methods were established prior to the conduct of the review and did the report justify any significant deviations from the protocol?** \| Yes \| \| --- \| --- \| |
|  |
| \| **3. Did the review authors explain their selection of the study designs for inclusion in the review?** \| No \| \| --- \| --- \| |
|  |
| \| **4. Did the review authors use a comprehensive literature search strategy?** \| Yes \| \| --- \| --- \| |
|  |
| \| **5. Did the review authors perform study selection in duplicate?** \| Yes \| \| --- \| --- \| |
|  |
| \| **6. Did the review authors perform data extraction in duplicate?** \| Yes \| \| --- \| --- \| |
|  |
| \| **7. Did the review authors provide a list of excluded studies and justify the exclusions?** \| Yes \| \| --- \| --- \| |
|  |
| \| **8. Did the review authors describe the included studies in adequate detail?** \| Yes \| \| --- \| --- \| |
|  |
| \| **9. Did the review authors use a satisfactory technique for assessing the risk of bias (RoB) in individual studies that were included in the review?** \| Yes \| \| --- \| --- \| \| **RCT** \|  \| \|  \|  \| |
| \| **10. Did the review authors report on the sources of funding for the studies included in the review?** \| Yes \| \| --- \| --- \| |
|  |
| \| **11. If meta-analysis was performed did the review authors use appropriate methods for statistical combination of results?** \|  \| \| --- \| --- \| \| **RCT** \| Yes \| \|  \|  \| |
|  |
| \| **12. If meta-analysis was performed, did the review authors assess the potential impact of RoB in individual studies on the results of the meta-analysis or other evidence synthesis?** \| No \| \| --- \| --- \| |
|  |
| \| **13. Did the review authors account for RoB in individual studies when interpreting/ discussing the results of the review?** \| Yes \| \| --- \| --- \| |
|  |
| \| **14. Did the review authors provide a satisfactory explanation for, and discussion of, any heterogeneity observed in the results of the review?** \| No \| \| --- \| --- \| |
|  |
| \| **15. If they performed quantitative synthesis did the review authors carry out an adequate investigation of publication bias (small study bias) and discuss its likely impact on the results of the review?** \| Yes \| \| --- \| --- \| |
|  |
| \| **16. Did the review authors report any potential sources of conflict of interest, including any funding they received for conducting the review?** \| Yes \| \| --- \| --- \| |

### Prevalence of vitamin D deficiency in South America: a systematic review and meta-analysis

Mendes MM, Gomes APO, Araújo MM, Coelho ASG, Carvalho KMB, Botelho PB. Prevalence of vitamin D deficiency in South America: a systematic review and meta-analysis. Nutrition Reviews. 2023:nuad010.

| \| Prevalence of vitamin D deficiency in South America: a systematic revi is a Moderate quality review \| \| \| --- \| --- \| \| **1. Did the research questions and inclusion criteria for the review include the components of PICO?** \| Yes \| \|  \| \| |
| --- | --- | --- | --- | --- | --- | --- |
| \| **2. Did the report of the review contain an explicit statement that the review methods were established prior to the conduct of the review and did the report justify any significant deviations from the protocol?** \| Yes \| \| --- \| --- \| |
|  |
| \| **3. Did the review authors explain their selection of the study designs for inclusion in the review?** \| Yes \| \| --- \| --- \| |
|  |
| \| **4. Did the review authors use a comprehensive literature search strategy?** \| Yes \| \| --- \| --- \| |
|  |
| \| **5. Did the review authors perform study selection in duplicate?** \| Yes \| \| --- \| --- \| |
|  |
| \| **6. Did the review authors perform data extraction in duplicate?** \| No \| \| --- \| --- \| |
|  |
| \| **7. Did the review authors provide a list of excluded studies and justify the exclusions?** \| Partial Yes \| \| --- \| --- \| |
|  |
| \| **8. Did the review authors describe the included studies in adequate detail?** \| Yes \| \| --- \| --- \| |
|  |
| \| **9. Did the review authors use a satisfactory technique for assessing the risk of bias (RoB) in individual studies that were included in the review?** \|  \| \| --- \| --- \| \|  \|  \| \| **NRSI** \| Yes \| |
|  |
| \| **10. Did the review authors report on the sources of funding for the studies included in the review?** \| No \| \| --- \| --- \| |
|  |
| \| **11. If meta-analysis was performed did the review authors use appropriate methods for statistical combination of results?** \|  \| \| --- \| --- \| \|  \|  \| \| **NRSI** \| Yes \| |
|  |
| \| **12. If meta-analysis was performed, did the review authors assess the potential impact of RoB in individual studies on the results of the meta-analysis or other evidence synthesis?** \| Yes \| \| --- \| --- \| |
|  |
| \| **13. Did the review authors account for RoB in individual studies when interpreting/ discussing the results of the review?** \| Yes \| \| --- \| --- \| |
|  |
| \| **14. Did the review authors provide a satisfactory explanation for, and discussion of, any heterogeneity observed in the results of the review?** \| Yes \| \| --- \| --- \| |
|  |
| \| **15. If they performed quantitative synthesis did the review authors carry out an adequate investigation of publication bias (small study bias) and discuss its likely impact on the results of the review?** \| Yes \| \| --- \| --- \| |
|  |
| \| **16. Did the review authors report any potential sources of conflict of interest, including any funding they received for conducting the review?** \| Yes \| \| --- \| --- \| |
|  |

### Effect of supplemental vitamin D3 on bone mineral density - a systematic review and meta-analysis

Kazemian E, Pourali A, Sedaghat F, Karimi M, Basirat V, Sajadi Hezaveh Z, et al. Effect of supplemental vitamin D3 on bone mineral density: a systematic review and meta-analysis. Nutrition reviews. 2022.

| \| Effect of supplemental vitamin D3 on bone mineral density: a systematic rev is a Low quality review \| \| \| --- \| --- \| \| **1. Did the research questions and inclusion criteria for the review include the components of PICO?** \| Yes \| \|  \| \| |
| --- | --- | --- | --- | --- | --- | --- |
| \| **2. Did the report of the review contain an explicit statement that the review methods were established prior to the conduct of the review and did the report justify any significant deviations from the protocol?** \| Yes \| \| --- \| --- \| |
|  |
| \| **3. Did the review authors explain their selection of the study designs for inclusion in the review?** \| No \| \| --- \| --- \| |
|  |
| \| **4. Did the review authors use a comprehensive literature search strategy?** \| Yes \| \| --- \| --- \| |
|  |
| \| **5. Did the review authors perform study selection in duplicate?** \| Yes \| \| --- \| --- \| |
|  |
| \| **6. Did the review authors perform data extraction in duplicate?** \| Yes \| \| --- \| --- \| |
|  |
| \| **7. Did the review authors provide a list of excluded studies and justify the exclusions?** \| Partial Yes \| \| --- \| --- \| |
|  |
| \| **8. Did the review authors describe the included studies in adequate detail?** \| Yes \| \| --- \| --- \| |
|  |
| \| **9. Did the review authors use a satisfactory technique for assessing the risk of bias (RoB) in individual studies that were included in the review?** \|  \| \| --- \| --- \| \| **RCT** \| Yes \| \|  \|  \| |
|  |
| \| **10. Did the review authors report on the sources of funding for the studies included in the review?** \| No \| \| --- \| --- \| |
|  |
| \| **11. If meta-analysis was performed did the review authors use appropriate methods for statistical combination of results?** \|  \| \| --- \| --- \| \| **RCT** \| Yes \| \|  \|  \| |
|  |
| \| **12. If meta-analysis was performed, did the review authors assess the potential impact of RoB in individual studies on the results of the meta-analysis or other evidence synthesis?** \| No \| \| --- \| --- \| |
|  |
| \| **13. Did the review authors account for RoB in individual studies when interpreting/ discussing the results of the review?** \| No \| \| --- \| --- \| |
|  |
| \| **14. Did the review authors provide a satisfactory explanation for, and discussion of, any heterogeneity observed in the results of the review?** \| Yes \| \| --- \| --- \| |
|  |
| \| **15. If they performed quantitative synthesis did the review authors carry out an adequate investigation of publication bias (small study bias) and discuss its likely impact on the results of the review?** \| Yes \| \| --- \| --- \| |
|  |
| \| **16. Did the review authors report any potential sources of conflict of interest, including any funding they received for conducting the review?** \| Yes \| \| --- \| --- \| |

## RANDOMIZED CONTROLED TRIALS

### The effect of monthly vitamin D supplementation on fractures: a tertiary outcome from the population-based, double-blind, randomised, placebo-controlled D-Health trial

Waterhouse M, Ebeling PR, McLeod DSA, et al. The effect of monthly vitamin D supplementation on fractures: a tertiary outcome from the population-based, double-blind, randomised, placebo-controlled D-Health trial. Lancet Diabetes Endocrinol. 2023;11(5):324-332.

| **Unique ID** | EV-1 | **Study ID** | D-Health 2023 | **Assessor** |  |
| --- | --- | --- | --- | --- | --- |
| **Ref or Label** | Waterhouse M, Ebeling PR, McLeod DSA, et al. The effect of monthly vitamin D supplementation on fractures: a tertiary outcome from the population-based, double-blind, randomised, placebo-controlled D-Health trial. Lancet Diabetes Endocrinol. 2023;11(5):324-332. | **Aim** | assignment to intervention (the 'intention-to-treat' effect) |  |  |
| **Experimental** | Vitamin D | **Comparator** | Placebo | **Source** | Journal article(s) |
| **Outcome** | Fractures | **Results** | 0.94 (0.84-1.06) | **Weight** | 1 |
| **Domain** | **Signalling question** | | | **Response** | **Comments** |
| **Bias arising from the randomization process** | 1.1 Was the allocation sequence random? | | | Y |  |
|  | 1.2 Was the allocation sequence concealed until participants were enrolled and assigned to interventions? | | | Y |  |
|  | 1.3 Did baseline differences between intervention groups suggest a problem with the randomization process? | | | NI |  |
|  | **Risk of bias judgement** | | | **Low** |  |
| **Bias due to deviations from intended interventions** | 2.1.Were participants aware of their assigned intervention during the trial? | | | N |  |
|  | 2.2.Were carers and people delivering the interventions aware of participants' assigned intervention during the trial? | | | N |  |
|  | 2.3. If Y/PY/NI to 2.1 or 2.2: Were there deviations from the intended intervention that arose because of the experimental context? | | | NA |  |
|  | 2.4 If Y/PY to 2.3: Were these deviations likely to have affected the outcome? | | | NA |  |
|  | 2.5. If Y/PY/NI to 2.4: Were these deviations from intended intervention balanced between groups? | | | NA |  |
|  | 2.6 Was an appropriate analysis used to estimate the effect of assignment to intervention? | | | Y |  |
|  | 2.7 If N/PN/NI to 2.6: Was there potential for a substantial impact (on the result) of the failure to analyse participants in the group to which they were randomized? | | | NA |  |
|  | **Risk of bias judgement** | | | **Low** |  |
| **Bias due to missing outcome data** | 3.1 Were data for this outcome available for all, or nearly all, participants randomized? | | | Y |  |
|  | 3.2 If N/PN/NI to 3.1: Is there evidence that result was not biased by missing outcome data? | | | NA |  |
|  | 3.3 If N/PN to 3.2: Could missingness in the outcome depend on its true value? | | | NA |  |
|  | 3.4 If Y/PY/NI to 3.3: Is it likely that missingness in the outcome depended on its true value? | | | NA |  |
|  | **Risk of bias judgement** | | | **Low** |  |
| **Bias in measurement of the outcome** | 4.1 Was the method of measuring the outcome inappropriate? | | | N |  |
|  | 4.2 Could measurement or ascertainment of the outcome have differed between intervention groups? | | | N |  |
|  | 4.3 Were outcome assessors aware of the intervention received by study participants? | | | N |  |
|  | 4.4 If Y/PY/NI to 4.3: Could assessment of the outcome have been influenced by knowledge of intervention received? | | | NA |  |
|  | 4.5 If Y/PY/NI to 4.4: Is it likely that assessment of the outcome was influenced by knowledge of intervention received? | | | NA |  |
|  | **Risk of bias judgement** | | | **Low** |  |
| **Bias in selection of the reported result** | 5.1 Were the data that produced this result analysed in accordance with a pre-specified analysis plan that was finalized before unblinded outcome data were available for analysis? | | | Y |  |
|  | 5.2 ... multiple eligible outcome measurements (e.g. scales, definitions, time points) within the outcome domain? | | | N |  |
|  | 5.3 ... multiple eligible analyses of the data? | | | N |  |
|  | **Risk of bias judgement** | | | **Low** |  |
| **Overall bias** | **Risk of bias judgement** | | | **Low** |  |

### Supplemental Vitamin D and Incident Fractures in Midlife and Older Adults

LeBoff MS, Chou SH, Ratliff KA, et al. Supplemental Vitamin D and Incident Fractures in Midlife and Older Adults. N Engl J Med. 2022;387(4):299-309

| **Unique ID** | **EV-2** | **Study ID** | **Vital 2022** | **Assessor** |  |
| --- | --- | --- | --- | --- | --- |
| **Ref or Label** | **LeBoff MS, Chou SH, Ratliff KA, et al. Supplemental Vitamin D and Incident Fractures in Midlife and Older Adults. N Engl J Med. 2022;387(4):299-309** | **Aim** | **assignment to intervention (the 'intention-to-treat' effect)** |  |  |
| **Experimental** | **Vitamin D** | **Comparator** | **Placebo** | **Source** | **Journal article(s); Trial protocol** |
| **Outcome** | **Fracture** | **Results** | **0.98 (0.89–1.08)** | **Weight** | **1** |
| **Domain** | **Signalling question** | | | **Response** | **Comments** |
| **Bias arising from the randomization process** | **1.1 Was the allocation sequence random?** | | | **Y** |  |
|  | **1.2 Was the allocation sequence concealed until participants were enrolled and assigned to interventions?** | | | **NI** |  |
|  | **1.3 Did baseline differences between intervention groups suggest a problem with the randomization process?** | | | **N** |  |
|  | **Risk of bias judgement** | | | **Some concerns** |  |
| **Bias due to deviations from intended interventions** | **2.1.Were participants aware of their assigned intervention during the trial?** | | | **N** |  |
|  | **2.2.Were carers and people delivering the interventions aware of participants' assigned intervention during the trial?** | | | **N** |  |
|  | **2.3. If Y/PY/NI to 2.1 or 2.2: Were there deviations from the intended intervention that arose because of the experimental context?** | | | **NA** |  |
|  | **2.4 If Y/PY to 2.3: Were these deviations likely to have affected the outcome?** | | | **NA** |  |
|  | **2.5. If Y/PY/NI to 2.4: Were these deviations from intended intervention balanced between groups?** | | | **NA** |  |
|  | **2.6 Was an appropriate analysis used to estimate the effect of assignment to intervention?** | | | **PY** |  |
|  | **2.7 If N/PN/NI to 2.6: Was there potential for a substantial impact (on the result) of the failure to analyse participants in the group to which they were randomized?** | | | **NA** |  |
|  | **Risk of bias judgement** | | | **Low** |  |
| **Bias due to missing outcome data** | **3.1 Were data for this outcome available for all, or nearly all, participants randomized?** | | | **Y** |  |
|  | **3.2 If N/PN/NI to 3.1: Is there evidence that result was not biased by missing outcome data?** | | | **NA** |  |
|  | **3.3 If N/PN to 3.2: Could missingness in the outcome depend on its true value?** | | | **NA** |  |
|  | **3.4 If Y/PY/NI to 3.3: Is it likely that missingness in the outcome depended on its true value?** | | | **NA** |  |
|  | **Risk of bias judgement** | | | **Low** |  |
| **Bias in measurement of the outcome** | **4.1 Was the method of measuring the outcome inappropriate?** | | | **N** |  |
|  | **4.2 Could measurement or ascertainment of the outcome have differed between intervention groups?** | | | **N** |  |
|  | **4.3 Were outcome assessors aware of the intervention received by study participants?** | | | **PN** |  |
|  | **4.4 If Y/PY/NI to 4.3: Could assessment of the outcome have been influenced by knowledge of intervention received?** | | | **NA** |  |
|  | **4.5 If Y/PY/NI to 4.4: Is it likely that assessment of the outcome was influenced by knowledge of intervention received?** | | | **NA** |  |
|  | **Risk of bias judgement** | | | **Low** |  |
| **Bias in selection of the reported result** | **5.1 Were the data that produced this result analysed in accordance with a pre-specified analysis plan that was finalized before unblinded outcome data were available for analysis?** | | | **Y** |  |
|  | **5.2 ... multiple eligible outcome measurements (e.g. scales, definitions, time points) within the outcome domain?** | | | **N** |  |
|  | **5.3 ... multiple eligible analyses of the data?** | | | **N** |  |
|  | **Risk of bias judgement** | | | **Low** |  |
| **Overall bias** | **Risk of bias judgement** | | | **Some concerns** |  |

### Effects of Supplemental Vitamin D on Bone Health Outcomes in Women and Men in the VITamin D and OmegA-3 TriaL (VITAL)

LeBoff MS, Chou SH, Murata EM, et al. Effects of Supplemental Vitamin D on Bone Health Outcomes in Women and Men in the VITamin D and OmegA-3 TriaL (VITAL). J Bone Miner Res. 2020;35(5):883-893. doi:10.1002/jbmr.3958

| **Unique ID** | EV-3 | **Study ID** | VITAL-BH | **Assessor** |  |
| --- | --- | --- | --- | --- | --- |
| **Ref or Label** | LeBoff MS, Chou SH, Murata EM, et al. Effects of Supplemental Vitamin D on Bone Health Outcomes in Women and Men in the VITamin D and OmegA-3 TriaL (VITAL). J Bone Miner Res. 2020;35(5):883-893. doi:10.1002/jbmr.3958 | **Aim** | assignment to intervention (the 'intention-to-treat' effect) |  |  |
| **Experimental** |  | **Comparator** |  | **Source** |  |
| **Outcome** |  | **Results** |  | **Weight** | 1 |
| **Domain** | **Signalling question** | | | **Response** | **Comments** |
| **Bias arising from the randomization process** | 1.1 Was the allocation sequence random? | | | Y |  |
|  | 1.2 Was the allocation sequence concealed until participants were enrolled and assigned to interventions? | | | Y |  |
|  | 1.3 Did baseline differences between intervention groups suggest a problem with the randomization process? | | | N |  |
|  | **Risk of bias judgement** | | | **Low** |  |
| **Bias due to deviations from intended interventions** | 2.1.Were participants aware of their assigned intervention during the trial? | | | N |  |
|  | 2.2.Were carers and people delivering the interventions aware of participants' assigned intervention during the trial? | | | N |  |
|  | 2.3. If Y/PY/NI to 2.1 or 2.2: Were there deviations from the intended intervention that arose because of the experimental context? | | | NA |  |
|  | 2.4 If Y/PY to 2.3: Were these deviations likely to have affected the outcome? | | | NA |  |
|  | 2.5. If Y/PY/NI to 2.4: Were these deviations from intended intervention balanced between groups? | | | NA |  |
|  | 2.6 Was an appropriate analysis used to estimate the effect of assignment to intervention? | | | Y |  |
|  | 2.7 If N/PN/NI to 2.6: Was there potential for a substantial impact (on the result) of the failure to analyse participants in the group to which they were randomized? | | | NA |  |
|  | **Risk of bias judgement** | | | **Low** |  |
| **Bias due to missing outcome data** | 3.1 Were data for this outcome available for all, or nearly all, participants randomized? | | | Y |  |
|  | 3.2 If N/PN/NI to 3.1: Is there evidence that result was not biased by missing outcome data? | | | NA |  |
|  | 3.3 If N/PN to 3.2: Could missingness in the outcome depend on its true value? | | | NA |  |
|  | 3.4 If Y/PY/NI to 3.3: Is it likely that missingness in the outcome depended on its true value? | | | NA |  |
|  | **Risk of bias judgement** | | | **Low** |  |
| **Bias in measurement of the outcome** | 4.1 Was the method of measuring the outcome inappropriate? | | | Y |  |
|  | 4.2 Could measurement or ascertainment of the outcome have differed between intervention groups? | | | N |  |
|  | 4.3 Were outcome assessors aware of the intervention received by study participants? | | | NA |  |
|  | 4.4 If Y/PY/NI to 4.3: Could assessment of the outcome have been influenced by knowledge of intervention received? | | | NA |  |
|  | 4.5 If Y/PY/NI to 4.4: Is it likely that assessment of the outcome was influenced by knowledge of intervention received? | | | NA |  |
|  | **Risk of bias judgement** | | | **Low** |  |
| **Bias in selection of the reported result** | 5.1 Were the data that produced this result analysed in accordance with a pre-specified analysis plan that was finalized before unblinded outcome data were available for analysis? | | | Y |  |
|  | 5.2 ... multiple eligible outcome measurements (e.g. scales, definitions, time points) within the outcome domain? | | | N |  |
|  | 5.3 ... multiple eligible analyses of the data? | | | N |  |
|  | **Risk of bias judgement** | | | **Low** |  |
| **Overall bias** | **Risk of bias judgement** | | | **Low** |  |

# EVIDENCE TABLES

**PICO**

Population: Healthy adults and the elderly residing in Latin America

Intervention: Vitamin D deficiency

Comparator:

| **Outcome**  Timeframe | **Study results and measurements** | **Absolute effect estimates** | | **Certainty of the Evidence**  (Quality of evidence) | **Plain language summary** |
| --- | --- | --- | --- | --- | --- |
|  |  | Comparator | Vitamin D deficiency |  |  |
| Prevalence of vitamin D deficiency^1^ |  |  | | **Very low**  Due to serious risk of bias^2^ | Of 9460 articles identified, 96 studies with a total of 227 758 participants were included. The overall prevalence of vitamin D deficiency was 34.76% (79 studies; 95%CI, 29.68–40.21; I2 = 99%). There were significant differences in prevalence rates related to age, sex, country, latitude, season, and year of publication. |

1. Prevalence of vitamin D deficiency (25-hydroxy-calciferol < 20 ng/mL) in Latin America
2. **Risk of Bias: serious.**

**References**

Mendes MM, Gomes APO, Araújo MM, Coelho ASG, Carvalho KMB, Botelho PB : Prevalence of vitamin D deficiency in South America: a systematic review and meta-analysis. Nutrition Reviews nuad010

**PICO**

Population: Community-dwelling adults with insufficiency

Intervention: Vitamin D treatment

Comparator: Placebo

| **Outcome**  Timeframe | **Study results and measurements** | **Absolute effect estimates** | | **Certainty of the Evidence**  (Quality of evidence) | **Plain language summary** |
| --- | --- | --- | --- | --- | --- |
|  |  | Placebo | Treatment |  |  |
| Any fracture^1^  1 year | Relative risk: 0.84  (CI 95% 0.58 - 1.21)  Based on data from 2186 participants in 6 studies^2^  Follow up 12 weeks to 3.5 years | **57**  per 1000 | **48**  per 1000 | **Moderate**  Due to serious imprecision^3^ | Treating vitamin D insufficiency in community-dwelling adults probably has little or no difference on any fracture |
|  |  | Difference: **9 fewer per 1000**  (CI 95% 24 fewer - 12 more) | |  |  |
| Hip fracture^4^  1 year | Relative risk: 0.86  (CI 95% 0.5 - 1.47)  Based on data from 3349 participants in 3 studies  Follow up 52 weeks to 3.5 years | **45**  per 1000 | **39**  per 1000 | **Low**  Due to serious risk of bias, Due to serious imprecision^5^ | Treating vitamin D insufficiency in community-dwelling adults probably has little or no difference on hip fracture |
|  |  | Difference: **6 fewer per 1000**  (CI 95% 22 fewer - 21 more) | |  |  |

1. The USPSTF systematic review assessed wheter treatment of community-dwelling adults with deficiency of vitamin D reduced the risk for any fractures.
2. Systematic review [16] . **Baseline/comparator** Control arm of reference used for intervention .
3. **Imprecision: serious.** due to study limitations on randomization and masquing.;
4. The USPSTF systematic review assessed wheter treatment of community-dwelling adults with deficiency of vitamin D reduced the risk for hip fractures.
5. **Risk of Bias: serious.** Inadequate sequence generation/ generation of comparable groups, resulting in potential for selection bias, Inadequate/lack of blinding of participants and personnel, resulting in potential for performance bias, Inadequate/lack of blinding of outcome assessors, resulting in potential for detection bias; **Imprecision: serious.** due to study limitations on randomization, masking and uncertainty in follow-up;

**References**

Kahwati LC, LeBlanc E., Weber RP, Giger K., Clark R., Suvada K., Guisinger A., Viswanathan M. : Screening for Vitamin D Deficiency in Adults: Updated Evidence Report and Systematic Review for the US Preventive Services Task Force. Jama 325(14):1443-1463

**PICO**

Population: Healthy premenopausal women

Intervention: Vitamin D

Comparator: Placebo

| **Outcome**  Timeframe | **Study results and measurements** | **Absolute effect estimates** | | **Certainty of the Evidence**  (Quality of evidence) | **Plain language summary** |
| --- | --- | --- | --- | --- | --- |
|  |  | Placebo | Vitamin D |  |  |
| Withdrawals from the study for any reason^1^  1 year | Relative risk: 0.74  (CI 95% 0.46 - 1.19)  Based on data from 189 participants in 2 studies^2^  Follow up median: 1 year | **233**  per 1000 | **172**  per 1000 | **Moderate**  Due to serious imprecision^3^ | Vitamin d probably has little or no difference on withdrawals from the study for any reason. |
|  |  | Difference: **61 fewer per 1000**  (CI 95% 126 fewer - 44 more) | |  |  |
| Lumbar spine BMD |  |  | |  | Vitamin D alone supplementation does not increase the BMD mean difference in the lumbar spine in healthy premenopausal woman. The studies included in these comparisons did not report the same outcomes and reported diKerent units of measure. |

1. Withdrawals for any reason
2. Systematic review [26] . **Baseline/comparator** Control arm of reference used for intervention .
3. **Risk of Bias: no serious.** Selective outcome reporting, Incomplete data and/or large loss to follow up, Inadequate/lack of blinding of outcome assessors, resulting in potential for detection bias; **Imprecision: serious.** Wide confidence intervals;

**References**

Méndez-Sánchez L., Clark P., Winzenberg TM, Tugwell P., Correa-Burrows P., Costello R. : Calcium and vitamin D for increasing bone mineral density in premenopausal women. The Cochrane database of systematic reviews 1(1):CD012664

**PICO**

Population: Adult men or women, > 20 years of age, without metabolic diseases affecting bone and calcium metabolism

Intervention: Vitamin D

Comparator: Placebo, no treatment, low dose vitamin D

| **Outcome**  Timeframe | **Study results and measurements** | **Absolute effect estimates** | | **Certainty of the Evidence**  (Quality of evidence) | **Plain language summary** |
| --- | --- | --- | --- | --- | --- |
|  |  | Control | Vitamin D |  |  |
| BMD at lumbar spine^1^  1 year | Measured by:  Scale: - High better  Based on data from 5957 participants in 34 studies^2^  Follow up 3 months to 5 years | Mean | Mean | **Low**  Due to serious risk of bias, Due to serious imprecision^3^ | Vitamin d may increase bmd at lumbar spine slightly |
|  |  | Difference: **SMD 0.06 higher**  (CI 95% 0.01 higher - 0.12 higher) | |  |  |
| BMD at femoral neck^4^  1 year | Measured by: DXA  Scale: - High better  Based on data from 6067 participants in 30 studies^5^  Follow up 3 months to 5 years | Mean | Mean | **Low**  Due to serious risk of bias, Due to serious imprecision, Due to serious inconsistency^6^ | Vitamin d probably increases bmd at femoral neck slightly |
|  |  | Difference: **SMD 0.25 higher**  (CI 95% 0.09 higher - 0.41 higher) | |  |  |
| BMD at total hip^7^  1 year | Measured by: DXA  Scale: - High better  Based on data from 5649 participants in 28 studies^8^  Follow up 3 months to 5 years | Mean | Mean | **Low**  Due to serious risk of bias, Due to serious inconsistency^9^ | Vitamin d may have little or no difference on bmd at total hip |
|  |  | Difference: **SMD 0.13 higher**  (CI 95% 0.03 lower - 0.29 higher) | |  |  |

1. Change in BMD at lumbar spine
2. Systematic review [18] . **Baseline/comparator** Control arm of reference used for intervention .
3. **Risk of Bias: serious.** Inadequate sequence generation/ generation of comparable groups, resulting in potential for selection bias, Inadequate concealment of allocation during randomization process, resulting in potential for selection bias, Inadequate/lack of blinding of participants and personnel, resulting in potential for performance bias, Inadequate/lack of blinding of outcome assessors, resulting in potential for detection bias, Incomplete data and/or large loss to follow up, Incomplete data and/or large loss to follow up; **Imprecision: serious.** Wide confidence intervals;
4. Change in BMD at femoral neck
5. Systematic review [18] . **Baseline/comparator** Control arm of reference used for intervention .
6. **Risk of Bias: serious.** Inadequate sequence generation/ generation of comparable groups, resulting in potential for selection bias, Inadequate concealment of allocation during randomization process, resulting in potential for selection bias, Inadequate/lack of blinding of participants and personnel, resulting in potential for performance bias, Inadequate/lack of blinding of outcome assessors, resulting in potential for detection bias, Incomplete data and/or large loss to follow up; **Inconsistency: serious.** The confidence interval of some of the studies do not overlap with those of most included studies/ the point estimate of some of the included studies., The magnitude of statistical heterogeneity was high, with I^2:... %.;
7. Change in BMD at total hip
8. Systematic review [18] . **Baseline/comparator** Control arm of reference used for intervention .
9. **Risk of Bias: serious.** Inadequate sequence generation/ generation of comparable groups, resulting in potential for selection bias, Inadequate concealment of allocation during randomization process, resulting in potential for selection bias, Inadequate/lack of blinding of participants and personnel, resulting in potential for performance bias, Inadequate/lack of blinding of outcome assessors, resulting in potential for detection bias, Incomplete data and/or large loss to follow up; **Inconsistency: serious.** The confidence interval of some of the studies do not overlap with those of most included studies/ the point estimate of some of the included studies., The magnitude of statistical heterogeneity was high, with I^2:... %.;

**References**

Kazemian E., Pourali A., Sedaghat F., Karimi M., Basirat V., Sajadi Hezaveh Z., Davoodi SH, Holick MF : Effect of supplemental vitamin D3 on bone mineral density: a systematic review and meta-analysis. Nutrition reviews

**PICO**

Population: Adults aged 60–84 years

Intervention: Vitamin D monthly doses of 60 000 IU

Comparator: Placebo

| **Outcome**  Timeframe | **Study results and measurements** | **Absolute effect estimates** | | **Certainty of the Evidence**  (Quality of evidence) | **Plain language summary** |
| --- | --- | --- | --- | --- | --- |
|  |  | Placebo | Vitamin D |  |  |
| Any fracture^1^  5 year | Hazard ratio: 0.94  (CI 95% 0.84 - 1.06)  Based on data from 20326 participants in 1 studies  Follow up 5 years | **59**  per 1000 | **56**  per 1000 | **High** | Vitamin d has little or no difference on any fracture |
|  |  | Difference: **3 fewer per 1000**  (CI 95% 9 fewer - 3 more) | |  |  |
| Non-vertebral fracture  5 year | Hazard ratio: 0.96  (CI 95% 0.85 - 1.08)  Based on data from 20326 participants in 1 studies  Follow up 5 years | **52**  per 1000 | **50**  per 1000 | **High** | Vitamin d has little or no difference on non-vertebral fracture |
|  |  | Difference: **2 fewer per 1000**  (CI 95% 8 fewer - 4 more) | |  |  |
| Major osteoporotic fracture  5 years | Hazard ratio: 1.0  (CI 95% 0.85 - 1.18)  Based on data from 20326 participants in 1 studies  Follow up 5 yeears | **29**  per 1000 | **29**  per 1000 | **High** | Vitamin d has little or no difference on major osteoporotic fracture |
|  |  | Difference: **0 fewer per 1000**  (CI 95% 4 fewer - 5 more) | |  |  |
| Hip fracture  5 years | Hazard ratio: 1.11  (CI 95% 0.86 - 1.45)  Based on data from 20326 participants in 1 studies  Follow up 5 years | **10**  per 1000 | **11**  per 1000 | **High** | Vitamin d has little or no difference on hip fracture |
|  |  | Difference: **1 more per 1000**  (CI 95% 1 fewer - 4 more) | |  |  |

1. First fracture at any site

**References**

Waterhouse M., Ebeling PR, McLeod DSA, English D., Romero BD, Baxter C., Armstrong BK, Hartel G., Kimlin M., O'Connell RL, van der Pols JC, Venn AJ, Webb PM, Whiteman DC, Neale RE : The effect of monthly vitamin D supplementation on fractures: a tertiary outcome from the population-based, double-blind, randomised, placebo-controlled D-Health trial. Lancet Diabetes Endocrinol 11(5):324-332

**PICO**

Population: Men 50 years of age or older and women 55 years of age or older

Intervention: vitamin D3 2000 IU per day

Comparator: Placebo

| **Outcome**  Timeframe | **Study results and measurements** | **Absolute effect estimates** | | **Certainty of the Evidence**  (Quality of evidence) | **Plain language summary** |
| --- | --- | --- | --- | --- | --- |
|  |  | Placebo | Vitamin D |  |  |
| Any fracture  5 years | Hazard ratio: 0.98  (CI 95% 0.89 - 1.08)  Based on data from 25871 participants in 1 studies  Follow up 5.3 years | **60**  per 1000 | **59**  per 1000 | **Moderate**  Due to serious risk of bias^1^ | Vitamin d probably has little or no difference on any fracture |
|  |  | Difference: **1 fewer per 1000**  (CI 95% 6 fewer - 5 more) | |  |  |
| Non-vertebral fracture  5 years | Hazard ratio: 0.97  (CI 95% 0.87 - 1.07)  Based on data from 25871 participants in 1 studies  Follow up 5.3 years | **57**  per 1000 | **55**  per 1000 | **Moderate**  Due to serious risk of bias^2^ | Vitamin d probably has little or no difference on non-vertebral fracture |
|  |  | Difference: **2 fewer per 1000**  (CI 95% 7 fewer - 4 more) | |  |  |
| Hip fracture  5 years | Hazard ratio: 1.01  (CI 95% 0.7 - 1.47)  Based on data from 25871 participants in 1 studies  Follow up 5.3 years | **4**  per 1000 | **4**  per 1000 | **Moderate**  Due to serious risk of bias^3^ | Vitamin d probably has little or no difference on hip fracture |
|  |  | Difference: **0 fewer per 1000**  (CI 95% 1 fewer - 2 more) | |  |  |
| Mean spine BMD at year 2  2 years | Measured by: DXA  Scale: - High better  Based on data from 771 participants in 1 studies  Follow up 2 | **1.03**  g/cm2Mean | **1.03**  g/cm2Mean | **High** | Vitamin d has little or no difference on change in spine bmd |
|  |  | Difference: **MD 0 lower** | |  |  |
| Mean femoral neck BMD at year 2  2 years | Measured by: DXA  Scale: - High better  Based on data from 771 participants in 1 studies  Follow up 2 years | **0.77**  g/cm2Mean | **0.77**  g/cm2Mean | **High** | Vitamin d has little or no difference on femoral neck bmd change |
|  |  | Difference: **MD 0.00 lower** | |  |  |

1. **Risk of Bias: serious.** Inadequate sequence generation/ generation of comparable groups, resulting in potential for selection bias;
2. **Risk of Bias: serious.** Inadequate sequence generation/ generation of comparable groups, resulting in potential for selection bias;
3. **Risk of Bias: serious.** Inadequate sequence generation/ generation of comparable groups, resulting in potential for selection bias;

**References**

LeBoff MS, Chou SH, Murata EM, Donlon CM, Cook NR, Mora S., Lee IM, Kotler G., Bubes V., Buring JE, Manson JE : Effects of Supplemental Vitamin D on Bone Health Outcomes in Women and Men in the VITamin D and OmegA-3 TriaL (VITAL). J Bone Miner Res 35(5):883-893

LeBoff MS, Chou SH, Ratliff KA, Cook NR, Khurana B., Kim E., Cawthon PM, Bauer DC, Black D., Gallagher JC, Lee IM, Buring JE, Manson JE : Supplemental Vitamin D and Incident Fractures in Midlife and Older Adults. N Engl J Med 387(4):299-309
